# Case report: Mononeuropathy multiplex of extranodal natural killer/T-cell lymphoma misdiagnosed as systemic vasculitis

**DOI:** 10.3389/fneur.2023.1283874

**Published:** 2023-11-21

**Authors:** Jiayu Shi, Jingwen Niu, Di Wu, Lei Zhang, Yangyu Huang, Hui Zhang, Hongzhi Guan, Mingsheng Liu, Yuzhou Guan

**Affiliations:** ^1^Neurology Department, Peking Union Medical College Hospital, Chinese Academy of Medical Sciences, Beijing, China; ^2^Rheumatology and Immunology Department, Peking Union Medical College Hospital, Chinese Academy of Medical Sciences, Beijing, China; ^3^Pathology Department, Peking Union Medical College Hospital, Chinese Academy of Medical Sciences, Beijing, China

**Keywords:** mononeuropathy multiplex, extranodal NK/T-cell lymphoma, axonal neuropathy, immunohistochemistry, biopsy

## Abstract

**Background:**

Extranodal NK/T-cell lymphoma (ENKTL) is an aggressive non-Hodgkin lymphoma that typically develops in the upper aerodigestive tract.

**Case presentation:**

We encountered an ENKTL patient who presented with purpura-like rashes and foot drops as initial symptoms and later developed other peripheral nerve involvement. The nerve conduction study of both the motor nerve and the sensory nerve showed axonal damage resembling mononeuropathy multiplex. Although the initial response to steroids was encouraging, the patient's symptoms reappeared and aggravated. A biopsy of the abdominal subcutaneous fat tissue with additional immunohistochemistry revealed neoplastic NK/T lymphocytes.

**Conclusion:**

We reported the first case presented as mononeuropathy multiplex as the initial clinical manifestation in ENKTL patients. Lymphoma should be considered in the diagnosis of atypical mononeuropathy in multiplex patients.

## Background

Extranodal NK/T-cell lymphoma (ENKTL) is an aggressive non-Hodgkin lymphoma (NHL) that most frequently occurs in East Asia and Latin America (1, 2). ENKTL usually arises in the nasal cavity, but extra-nasal sites are sometimes involved, including the skin, soft tissue, and gastrointestinal tract (3). Extra-nasal site involvement might cause infrequent manifestations. The morphology and immunohistochemical characteristics of neoplastic cells are various. Since the clinical and pathological variability of ENKTL can sometimes be misleading, the correct diagnosis of patients might be delayed. ENKTL is scarcely relevant to PNS manifestations. We here report a case of ENKTL, which first manifested as mononeuropathy multiplex mimicking systemic vasculitis and gradually developed polyneuropathy.

## Case presentation

**Figure 1:**
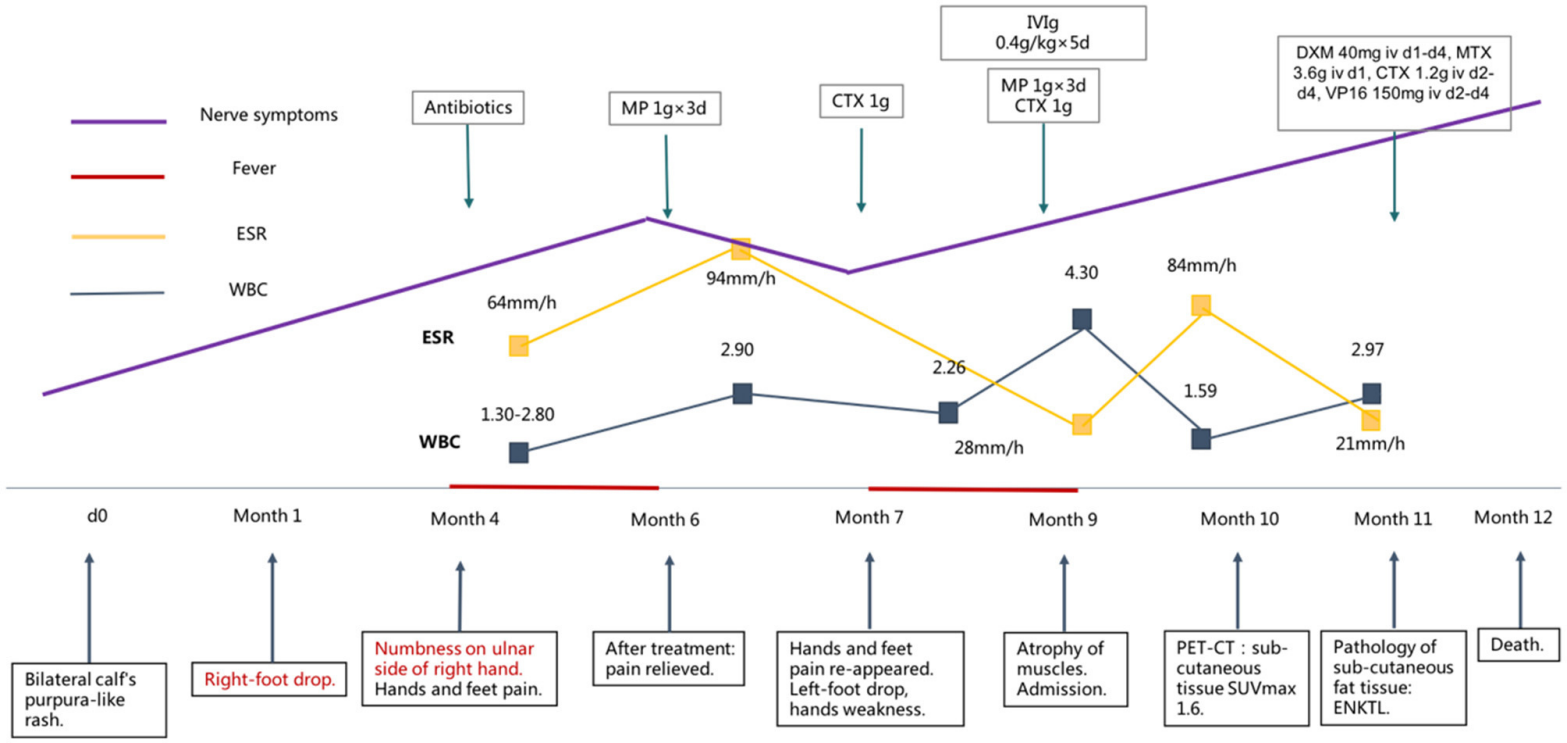
Clinical and therapeutic course of the patient. ENKTL, extranodal NK/T-cell lymphoma, MP, methylprednisolone, CTX, cytoxan, IVIg, intravenous immunoglobulins, DXM, dexamethasone, MTX, methotrexate, VP16, vepeside, PET-CT, positron emission tomography/computed tomography.

An 18-year-old previously healthy female experienced bilateral purpura-like rashes on both calves 9 months before admission. The details of the subsequent clinical course are outlined in timeline form in Figure 1. After 1 month, she developed a sudden right-foot drop and steppage gait. On month 4, she noticed numbness on the ulnar side of her right hand with an intermittent fever of 39°C. She was admitted to a local hospital, and an abnormal full blood count was found (WBC 1.30–2.9 × 10^9/^L, Hgb 91–95 g/L, PLT 175–238 × 10^9^/L). ESR was elevated to 64–94 mm/h. Serum immunoglobulin G (IgG) was 37.62 g/L. Autoimmune antibodies provided a positive result for PR3-anti-neutrophil cytoplasmic antibody (ANCA). Urine routine, liver and kidney function, serum Epstein–Barr virus (EBV), and cytomegalovirus (CMV) DNA, high-sensitive C-reactive protein (hsCRP), serum immunofixation electrophoresis, bone marrow biopsy, bone marrow pathology, and bone marrow flow cytometry analysis showed no abnormality. Nerve conduction studies were conducted, and the results are shown in Supplementary Table 1. Cranial, cervical, and thoracic vertebrae magnetic resonance imaging (MRI) showed no obvious abnormality. She was then treated with antibiotics, and her fever was relieved, but in month 5, she gradually developed symptoms of bilateral pain in both hands and feet, which disturbed her daily life. She was diagnosed with systemic vasculitis and was referred to the Department of Rheumatology and Immunology for further diagnosis. Serum tests showed PR3-ANCA at 60 RU/ml titer and anti-nuclear antibody (ANA) S1:160(+). She was diagnosed with connective tissue disease and was treated with methylprednisolone (MP) 1 g intravenously for 3 days, followed by 48 mg prednisone orally. She was also treated with cytoxan (CTX) 1 g intravenously. After treatment, her symptoms of hand and foot pain were relieved dramatically, but limb weakness and numbness did not change. On month 7, hand and foot pain re-appeared to a severe extent, and she developed a sudden left-foot drop. She gradually lost the strength of her left hand, and on month 9, she was unable to hold a spoon or lift her both wrists. She was treated with MP 1 g × 3 days, CTX 1 g once, and intravenous immunoglobulins (IVIg) at 0.4 g/kg × 10 days, but her symptoms did not relieve. On month 9, muscle atrophy could be observed on both of her hands, forearms, and calves. Before 1 week of admission, she developed signs of left peripheral facial palsy. She was admitted to our hospital 9 months after the disease onset.

Physical examinations at admission showed a few old eruption stains on both calves, but no fresh rashes could be observed. Nervous system physical examinations showed signs of left peripheral facial palsy. Tendon reflexes were absent. The muscle atrophy of the thenar and interosseous muscles of both hands could be observed. The proximal muscle strength of the right upper limb is grade IV, while the left upper limb is grade III. The strength of the distal muscles of the upper limbs was grades I–II. The proximal muscle strength of both lower limbs is grade V, and the muscle strength of the dorsum of the foot is grades I–II. The muscle tension was normal. The Romberg sign was positive. The sensation of acupuncture and vibration decreased in a sock-like and glove-like fashion in both upper and lower limbs.

**Figure 2:**
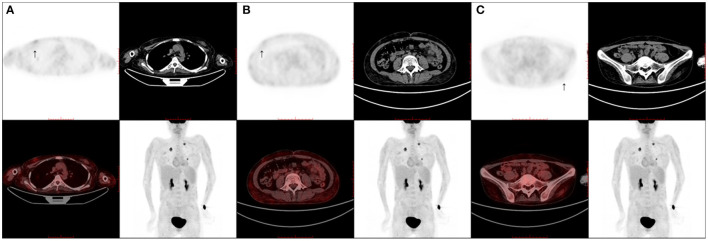
Positron emission tomography/computed tomography scans. A positron emission tomography/computed tomography scan shows multiple slightly fluorodeoxyglucose-avid subcutaneous fat tissue lesions scattered throughout the body (SUVmax of 1.6). **(A)** Thoracic wall (arrow); **(B)** abdominal wall (arrow); **(C)** pelvic wall (arrow).

The work-up, including serum tests, cerebral-spinal fluid (CSF) examinations, positron emission tomography-computer tomography (PET-CT), and electrodiagnostic tests, was conducted after admission. Serum tests for EBV DNA showed a positive result with a copy number varying from 500 to 2,400/ml. Serum tests for IgG and IgM of HBV, HCV, HIV, RPR, TPPA, CMV, Lyme, and brucellosis were all negative. Serum tests of ANA, ANCA, and anti-C1q were negative. Serum and CSF anti-GM1, anti-GQ1b, anti-Hu, anti-Yo, anti-Ri, anti-α-amino-3-hydroxy-5-methyl-4-isoxazole-propionicacid (AMPA), anti-CV2, anti-γ-aminobutyric acid (GABA), anti-Amphiphysin, anti-contactin-associated protein 2 (CASPR2), and anti-leucine-rich glioma-inactivated 1 (LGI1) were all negative. The serum levels of interleukin (IL) 10 and tumor necrosis factor (TNF) α were elevated at 16.4 and 28.5 pg/ml. The CSF results showed a slightly elevated protein level of 52–54 mg/dL, and the cell count was 32–34 cells/μl. Oligoclonal bands were negative in CSF. Cells in CSF were mostly lymphocytes, and no atypical cell was observed. Next-generation sequencing (NGS) for pathogens in CSF proved to be positive for EBV with a copy number of 2,564. The CSF levels of IL6, IL8, and IL10 were all elevated at 18.3, 222, and 15.4 pg/ml, respectively. CSF flow cytometry was negative. PET-CT gave a very interesting finding of hypermetabolism in the subcutaneous fat tissue all over the body with a slightly elevated standard uptake value (SUV) maximum of 1.6 (Figure 2). PET-CT also showed a soft tissue signal with a SUVmax of 3.3 in the bilateral nasal meatus. Nerve conduction study results are shown in Supplementary Table 1. Electromyogram (EMG) results showed an enormous amount of positive sharp waves and fibrillation discharges in the left deltoideus muscle and left tibialis anterior muscle. Nerve ultrasound was conducted and revealed an unremarkable cross-sectional area (CSA) enlargement in bilateral median, posterior tibial, and peroneal nerves.

**Figure 3:**
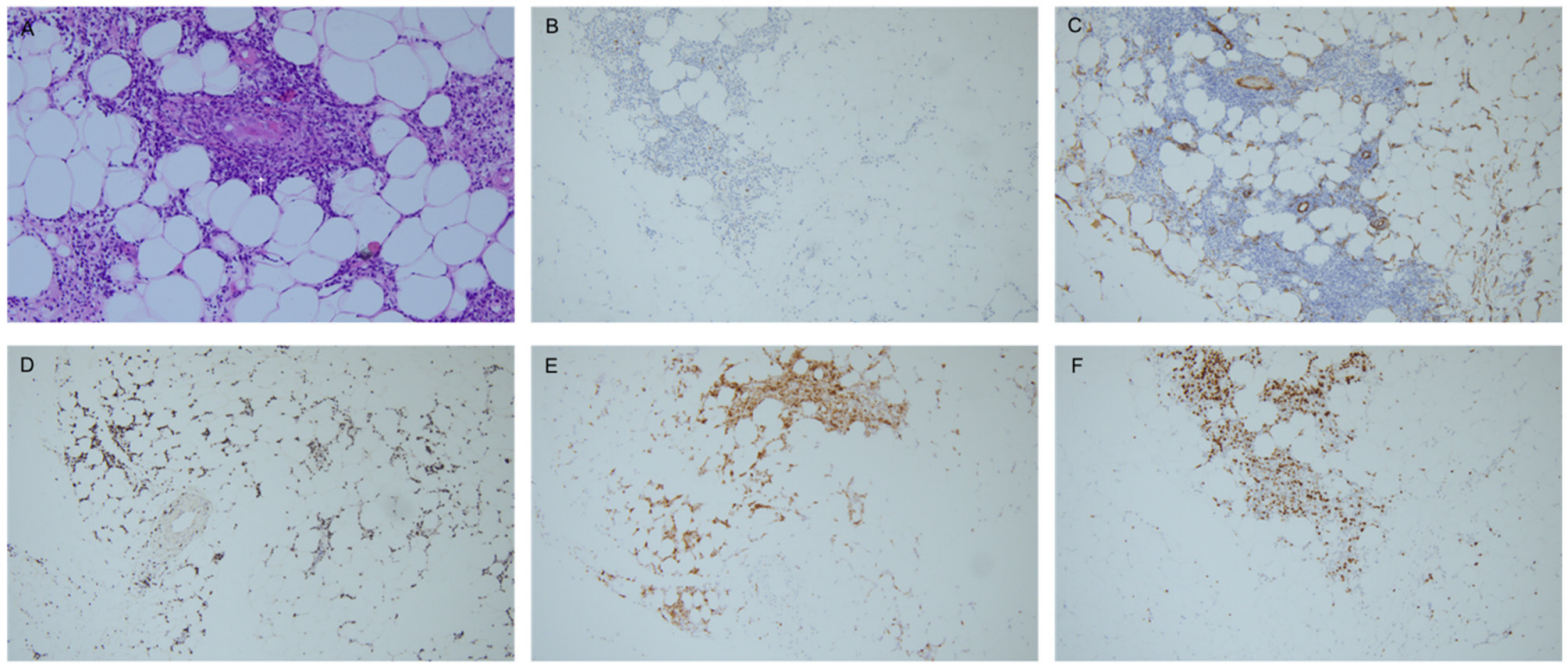
Immunohistochemical staining. **(A)** Histological findings of the subcutaneous fat tissue (hematoxylin-eosin: **A**, × 200), showing distended vessels filled with atypically large lymphoid cells, causing vessel occlusion (arrow). Infiltration of atypical lymphoid cells could also be observed in the subcutaneous fat tissue. **(B–E)** Immunohistochemical staining was positive for CD20 (**B**, × 100), CD34 (**C**, × 100), EBER (**D**, × 100), granzyme B (**E**, × 100). F: Ki-67 index 80% (**F**, × 100). Pathological figures are provided by the Pathology Department, Peking Union Medical College Hospital.

Pathological tests of both abdominal subcutaneous fat tissue and left nasal meatus soft tissue were conducted subsequently. The histologic study of abdominal subcutaneous fat tissue revealed many distended vessels filled with atypically large lymphoid cells, causing vessel occlusion. The tumor cells had large, irregular hyperchromatic nuclei and ample eosinophilic cytoplasm that was not only confined to the vessels but also disseminated in the fat tissue. Immunohistochemical staining showed the cluster of differentiation (CD)21(–), CD138(–), CD34(around vessels+), CD20(+), CD3(+), Ki-67(index 80%), CD56(–), CD5(+), CD7(-), CD2(+), CD4(+), CD38(+), granzyme B(+), TIA-1(+), and EBER (+), which consisted of the diagnosis of ENKTL (Figure 3). The left nasal meatus soft tissue showed a microscopic finding of pseudostratified ciliated columnar epithelium infiltrated by lymphocytes and plasma cells, which was regarded as an EBV-related T-cell proliferative disease. The patient was diagnosed as ENKTL, staging IVb.

The patient was then treated with a classic scheme for ENKTL, including dexamethasone (DXM) 40 mg iv d1-d4, methotrexate (MTX) 3.6 g iv d1, cytoxan (CTX) 1.2 g iv d2-d4, vepeside (VP16) 150 mg iv d2-d4, after which her nervous system symptoms showed no improvement. The patient developed septic shock and digestive tract hemorrhage and succumbed to the disease 12 months after its disease onset.

## Discussion and conclusion

Lymphomas are hematopoietic neoplasms originating from immunocompetent cells. They are divided into the NHL and HL (1). HL and NHL both infrequently cause peripheral nervous system complications, with NHL a much more common cause. In a case series, 98% of lymphoma patients with peripheral nerve system complications had NHL (2, 3). Most are B-cell NHL, with T-cell NHL accounting for only approximately 10% (2). The mechanisms of nerve damage caused by lymphoma are usually not clear, but several hypotheses have been proposed. First, direct invasion of lymphoma cells into the PNS, known as neurolymphomatosis is the most common cause of PNS system involvement (1, 4). Malignant lymphocytes that react with the antibody of a neural cell adhesion molecule, including CD56, may adhere to neural cell adhesion molecules, which allow the passage of certain classes of lymphocytes across the blood–nerve barrier, causing neurolymphomatosis (5). Most infiltrative neuropathies in humans are due to B-cell lymphoma, but peripheral T-cell lymphoma has also been reported to cause infiltrative neuropathies (1, 6). This phenomenon may cause unusual sites of involvement in lymphoma. Second, inflammatory, dys-immune neuropathies such as GBS, CIDP, or vasculitic neuropathy can occur in lymphoma due to the accompanying or preexisting immune perturbation, also known as paraneoplastic neuropathy (4, 7). These disorders are more commonly reported with HL than NHL. Third, hematogenous metastases can occlude vessels by local intravascular proliferation or direct pressure (8). Angiotrophic lymphoma, characterized by neoplastic lymphoid cells and immune-complex deposition within the walls of the vasa nervorum, could lead to vessel occlusion and cause nerve infarction (9).

ENKTL is most common in Asia and Central and South America and affects adults with a male predominance. It classically arises in the nasal cavity, the paranasal sinuses, or the palate and is associated with EBV infection but can also be observed in 15–20% of other sites such as the skin (60%), sub-cutaneous tissue, gastro-intestinal tract, testis, and even pancreas, without nasal localization (10). ENKTL is scarcely related to peripheral nervous system complications, based on our review of the English literature (Table 1), and only six cases of ENKTL invasion of peripheral nerves have been reported. All cases reported are women, and the age range is from 17 to 70 years. Most cases' clinical presentation is mononeuropathy, presented as tumor infiltration of a single nerve, including the median nerve, ulnar nerve, and tibial nerve (5, 11, 12). In addition to our case, two other cases presented as polyneuropathy (13, 14). One case, reported by Tian et al., (13) also presented with rashes before nervous system involvement. In another case, because of nerve root enlargement, AIDP was diagnosed in the first place (14).

**Table 1:** Clinical characteristics of patients with ENKTCL-NT combined with peripheral neuropathy.

**References**	**Year**	**Age/sex**	**Neuropathy manifestation**	**Primary site of ENKTL**	**Primary diagnosis**	**Prognosis**
Kim et al. (5)	1998	70/F	Mononeuropathy, median nerve	NR	NR	Survival
Wills et al. (14)	2008	29/F	Lumbar nerve roots	Lumbar nerve roots	AIDP	Survival
Agrawal et al. (11)	2013	57/F	Mononeuropathy, ulnar nerve	Nasal cavity	ENKTL	Survival
Aynardi et al. (12)	2018	59/F	Mononeuropathy, tibial nerve	Nasal cavity	ENKTL	Survival
Tian et al. (13)	2023	17/F	Polyneuropathy	Skin	Granuloma annulare	Survival
Our case	2023	18/F	Mononeuropathy multiplex	Skin	Vasculitis	Death

NR, not recorded.

Back to our case, it is a pity that the patients' parents refused a nerve biopsy, worrying about the deterioration of nerve function caused by the procedure. As a nerve biopsy was not obtainable, the mechanism of patient PNS involvement could only be hypothesized. We conducted nerve ultrasonography to directly observe the CSA of involved nerves, showing no obvious CSA enlargement that does not support neurolymphomatosis (15). From the abdominal subcutaneous fat tissue biopsy, we could observe vessel occlusions and vessel wall thickening (Figure 3A, arrow), which pathologically support the hypothesis of nerve infarction caused by vessel occlusions. Additionally, the patient's tumor cells expressed the antibody CD34 (Figure 3C), which is reactive to the vascular endothelial cell marker, leading to tumor cells propensity to attach to vessel walls (16). In contrast with typical ENKTL, the immunohistochemical result of our case is relatively rare since CD56 was negative while CD20 was positive. Although CD56 is an important clue to diagnose NK/T cell lymphoma and is believed to be a marker for ENKTL, it has been infrequently reported to be negative in some cases, especially in cases with uncommon clinical manifestations (17). ENKTL with aberrant CD20 expression is extremely rare. To the best of our knowledge, there have only been 11 CD20-positive ENKTLs reported. ENKTL with aberrant CD20 expression primarily occurred at extra-nasal sites rather than in the nasal cavity, including the testis, skin, and soft tissue, with a relatively aggressive clinical course, which is in accordance with our case (18). The rare immunohistochemistry of our case could help to explain infrequent clinical symptoms and the site of tumor origin. The patient's original diagnosis was systematic vasculitis, which was based on the result of a positive PR3 ANCA in the former hospital. The false-positive result of auto-antibody is not uncommon in lymphoma patients due to cross-immune reactions (13) and could be misleading in these patients' diagnoses.

In conclusion, we reported that the first case manifested as mononeuropathy multiplex and was diagnosed as ENKTL. Patients with lymphoma can manifest various neuropathic patterns. Although an infrequent clinical manifestation, lymphoma should never be overlooked in patients with neuropathy of various forms, especially those with mononeuropathy multiplex. It is possible that mononeuropathy multiplex is misdiagnosed as systematic angiitis since the clinical course could be similar and show the presence of signs of an axonal neuropathy pattern. ENKTL is extremely rare to show PNS symptoms, and its mechanism of neuropathy is still not established, requiring further research.

## Data availability statement

The raw data supporting the conclusions of this article will be made available by the authors, without undue reservation.

## Ethics statement

The studies involving humans were approved by Peking Union Medical College Hospital. The studies were conducted in accordance with the local legislation and institutional requirements. The human samples used in this study were acquired from a by-product of routine care or industry. Written informed consent for participation was not required from the participants or the participants' legal guardians/next of kin in accordance with the national legislation and institutional requirements. Written informed consent was obtained from the individual(s) for the publication of any potentially identifiable images or data included in this article.

## Author contributions

JS: Writing—original draft, Writing—review & editing, Conceptualization, Methodology, Supervision. JN: Conceptualization, Methodology, Writing—review & editing. DW: Conceptualization, Investigation, Supervision, Writing—review & editing. LZ: Methodology, Writing—review & editing. YH: Data curation, Methodology, Writing—review & editing. HZ: Methodology, Writing—review & editing. HG: Conceptualization, Supervision, Writing—review & editing. ML: Methodology, Supervision, Writing—review & editing. YG: Conceptualization, Methodology, Supervision, Writing—review & editing.

## References

[1] Kelly JJ Karcher DS. Lymphoma and peripheral neuropathy: a clinical review. Muscle Nerve. (2005) 31:301–13. 10.1002/mus.20163 15543550

[2] Giglio P Gilbert MR. Neurologic complications of non-Hodgkin's lymphoma. Curr Oncol Rep. (2005) 7:61–5. 10.1007/s11912-005-0027-8 15610688

[3] Diaz-Arrastia R Younger DS Hair L Inghirami G Hays AP Knowles DM. Neurolymphomatosis: a clinicopathologic syndrome re-emerges. Neurology. (1992) 42:1136–41. 10.1212/wnl.42.6.1136 1340762

[4] Tomita M Koike H Kawagashira Y Iijima M Adachi H Taguchi J. Clinicopathological features of neuropathy associated with lymphoma. Brain. (2013) 136(Pt 8):2563–78. 10.1093/brain/awt193 23884813

[5] Kim J Kim YS Lee EJ Kang CS Shim SI. Primary CD56-positive NK/T-cell lymphoma of median nerve: a case report. J Korean Med Sci. (1998) 13:331–3. 10.3346/jkms.1998.13.3.331 9681817 PMC3054500

[6] Kern WF Spier CM Hanneman EH Miller TP Matzner M Grogan TM. Neural cell adhesion molecule-positive peripheral T-cell lymphoma: a rare variant with a propensity for unusual sites of involvement. Blood. (1992) 79:2432–7. 1373974

[7] Kuchukhidze G Helbok R Unterberger I Koppelstaetter F Bodner T Trinka E. Bilateral mesial temporal polymicrogyria: a case report. J Neurol Neurosurg Psychiatry. (2008) 79:483–4. 10.1136/jnnp.2007.138792 18344402

[8] Vital C Vital A Julien J Rivel J deMascarel A Vergier B. Peripheral neuropathies and lymphoma without monoclonal gammopathy: a new classification. J Neurol. (1990) 237:177–85. 10.1007/bf00314591 2164577

[9] Lynch KM Katz JD Weinberg DH Lin DI Folkerth RD. Isolated mononeuropathy multiplex–a rare manifestation of intravascular large B-cell lymphoma. J Clin Neuromuscul Dis. (2012) 14:17–20. 10.1097/CND.0b013e318262ab5c 22922577

[10] Dotlic S Ponzoni M King RL Oschlies I Ferry J Calaminici M. The broad and challenging landscape of extranodal lymphoproliferations. Virchows Arch. (2020) 476:633–46. 10.1007/s00428-019-02702-w 31758317

[11] Agrawal S Gi MT Ng SB Puhaindran ME Singhania P. MRI and PET-CT in the diagnosis and follow-up of a lymphoma case with multifocal peripheral nerve involvement. Diagn Interv Radiol. (2013) 19:25–8. 10.4261/1305-3825.DIR.5876-12.1 23255069

[12] Aynardi M Raikin SM. Recurrence of extranodal natural killer/T-cell lymphoma presenting as tarsal tunnel syndrome. Am J Orthop. (2018) 47. 10.12788/ajo.2018.0025 29883499

[13] Tian Z Tian J Liao J. NK/T-cell lymphoma with rash and peripheral neuropathy as the first manifestation: a case report and literature review. Diagn Pathol. (2023) 18:2. 10.1186/s13000-023-01286-z 36627681 PMC9830812

[14] Wills AJ O'Connor S McMillan A. Sub-acute demyelinating neuropathy associated with an NK/T cell lymphoma. J Neurol Neurosurg Psychiatry. (2008) 790:484–5. 10.1136/jnnp.2007.133694 18344403

[15] Kerasnoudis A Tsivgoulis G. Nerve ultrasound in peripheral neuropathies: a review. J Neuroimaging. (2015) 25:528–38. 10.1111/jon.12261 25996962

[16] Liu Y Zhang W An J Li H Liu S. Cutaneous intravascular natural killer-cell lymphoma: a case report and review of the literature. Am J Clin Pathol. (2014) 142:243–7. 10.1309/AJCP1JLYXLGDNOCH 25015867

[17] Liu LH Huang Q Liu YH Yang J Fu H Jin L. Muscular involvement of extranodal natural killer/T cell lymphoma misdiagnosed as polymyositis: a case report and review of literature. World J Clin Cases. (2020) 8:963–70. 10.12998/wjcc.v8.i5.963 32190634 PMC7062622

[18] Huang Y Chen S Wei R Guo X Yang X Cao Q. CD20-positive extranodal NK/T cell lymphoma: clinicopathologic and prognostic features. Virchows Arch. (2020) 477:873–83. 10.1007/s00428-020-02776-x 32314054

