# Supplementary material for: Case report: Mononeuropathy multiplex of extranodal natural killer/T-cell lymphoma misdiagnosed as systemic vasculitis
Source: Front Neurol. 2023 Nov 21;14:1283874. doi: 10.3389/fneur.2023.1283874 (PMC10702496; doi:10.3389/fneur.2023.1283874)
Supplement: Supplementary file 1 [file Table_1.DOCX]

|  | 2020-07 | | | | 2020-09 | | | |
| --- | --- | --- | --- | --- | --- | --- | --- | --- |
|  | Motor Nerve | | Sensory Nerve | | Motor Nerve | | Sensory Nerve | |
|  | CV (m/s) | CMAP (mV) | CV (m/s) | SNAP (μV) | CV (m/s) | CMAP (mV) | CV (m/s) | SNAP (μV) |
| Right median | 54 | 7.064 | 51 | 42.79 | NR | NR | NR | NR |
| Right ulnar | 42 | 3.708 | N/A | N/A | NR | NR | NR | NR |
| Right peroneal | NR | 0.787 | 34 | 1.143 | NR | NR | NR | NR |
| Right tibial | 38 | 7.611 | N/A | N/A | NR | NR | NR | NR |
| Left peroneal | 34 | 1.014 | N/A | N/A | NR | 0.45 | NR | NR |
| Left tibial | 40 | 10.09 | 37 | 1.221 | NR | NR | NR | NR |
| Left median | 53 | 9.557 | 54 | 10.91 | 49.6/51.6^‡^ | 2.6 | 46.5/48.1^§^ | 0.99 |
| Left ulnar | 47 | 6.92 | N/A | N/A | NR | NR | NR | NR |
| Left peronaeus superficialis | N/A | N/A | 41 | 2.475 | NR | NR | NR | NR |
| Right peronaeus superficialis | N/A | N/A | 43 | 3.498 | NR | NR | NR | NR |
| Left sural | N/A | N/A | 50 | 12.17 | NR | NR | NR | NR |
| Right sural | N/A | N/A | 47 | 1.073 | NR | NR | NR | NR |
| Left radial | N/A | N/A | N/A | N/A | NR | NR | NR | NR |
| Right radial | N/A | N/A | 48 | 9.235 | NR | NR | NR | NR |

Supplementary Table 1. Nerve conduction results of the patient in July, 2020 and September, 2020. Median motor response stimulating at the wrist/antecubital fossa.‡Median motor conduction velocities from wrist to elbow/axillia.§Median sensory velocities from digitalis I/digitalis III to wristat the medial ankle/popliteal fossa.Abbreviations: SNAP, sensory nerve action potential; CMAP, compound muscle action potential; CV, conduction velocity; NR, no response; N/A, not performed;
